# Engagement in Primary Prevention Program among Rural Veterans With Osteoporosis Risk

**DOI:** 10.1002/jbm4.10682

**Published:** 2022-10-03

**Authors:** Karla L. Miller, Kimberly Mccoy, Chris Richards, Aaron Seaman, Samantha L. Solimeo

**Affiliations:** ^1^ VHA Office of Rural Health, Veterans Rural Health Resource Center‐Salt Lake City, Department of Internal Medicine, Rheumatology Section Veterans Affairs Salt Lake City Health Care System Salt Lake City Utah USA; ^2^ Associate Professor (Clinical) of Medicine, Division of Rheumatology University of Utah School of Medicine Salt Lake City Utah USA; ^3^ VHA Office of Rural Health, Veterans Rural Health Resource Center‐Iowa City (VRHRC‐IC), Center for Access & Delivery Research and Evaluation (CADRE) Veterans Affairs Iowa City VHA Health Care System Iowa City Iowa USA; ^4^ VHA Office of Rural Health, Veterans Rural Health Resource Center‐Iowa City (VRHRC‐IC), Center for Access & Delivery Research and Evaluation (CADRE) Department of Veterans Affairs Iowa City VHA Health Care System Iowa City Iowa USA; ^5^ VHA Office of Rural Health, Veterans Rural Health Resource Center‐Iowa City (VRHRC‐IC) Veterans Affairs Iowa City VHA Health Care System Iowa City Iowa USA; ^6^ Division of General Internal Medicine, Department of Internal Medicine, Carver College of Medicine University of Iowa Iowa City Iowa USA; ^7^ VHA Office of Rural Health, Veterans Rural Health Resource Center‐Iowa City (VRHRC‐IC), Center for Access & Delivery Research and Evaluation (CADRE), Primary Care Analytics Team Iowa City (PCAT‐IC) Veterans Affairs Iowa City VHA Health Care System Iowa City Iowa USA; ^8^ Division of General Internal Medicine, Department of Internal Medicine, Carver College of Medicine University of Iowa Iowa City Iowa USA

**Keywords:** DXA, FRACTURE PREVENTION, HEALTH SERVICES RESEARCH, OSTEOPOROSIS, SCREENING

## Abstract

A primary osteoporosis prevention program using a virtual bone health team (BHT) was implemented to comanage the care of rural veterans in the Mountain West region of the United States. The BHT identified, screened, and treated rural veterans at risk for osteoporosis using telephone and United States Postal Service communications. Eligibility was determined by regular use of Veterans Health Administration primary care, age 50 or older, and evidence of fracture risk. This study was conducted to identify demographic and clinical factors associated with the acceptance of osteoporosis screening and the initiation of medication where indicated. A cross‐sectional cohort design (*N* = 6985) was utilized with a generalized estimating equation and logit link function to account for facility‐level clustering. Fully saturated and reduced models were fitted using backward selection. Less than a quarter of eligible veterans enrolled in BHT's program and completed screening. Factors associated with a lower likelihood of clinic enrollment included being of older age, unmarried, greater distance from VHA services, having a copayment, prior fracture, or history of rheumatoid arthritis. A majority of veterans with treatment indication started medication therapy (*N* = 453). In this subpopulation, Fisher's exact test showed a significant association between osteoporosis treatment uptake and a history of two or more falls in the prior year, self‐reported parental history of fracture, current smoking, and weight‐bearing exercise. The BHT was designed to reduce barriers to screening; however, for this population cost and travel continue to limit engagement. The remarkable rate of medication initiation notwithstanding, low enrollment reduces the impact of this primary prevention program, and findings pertaining to fracture, smoking, and exercise imply that health beliefs are an important contributing factor. Efforts to identify and address barriers to osteoporosis screening and treatment, such as clinical factors, social determinants of health, and health beliefs, may pave the way for effective implementation of population bone health care delivery systems. Published 2022. This article is a U.S. Government work and is in the public domain in the USA. *JBMR Plus* published by Wiley Periodicals LLC on behalf of American Society for Bone and Mineral Research.

## Introduction

Fragility fractures are the most serious and costly complication of osteoporosis, which affects nearly 10 million people in the United States, including 2 million men.^(^ ^1^ ^)^ Risk identification, screening, and treatment are critical elements of osteoporosis and fragility fracture prevention, but underrecognition of risk, underuse of dual‐energy X‐ray absorptiometry (DXA) screening, undertreatment of those at highest risk, and poor medication adherence contribute to a worrisome gap between best practice and current practice.^(^ ^2^, ^3^, ^4^ ^)^ Multidisciplinary Fracture Liaison Service (FLS) models of care have been among the most successful and cost‐effective interventions for secondary fracture prevention,^(^ ^5^, ^6^ ^)^ but implementation of primary prevention to reduce the number of first fractures remains a challenge, in part because risk identification relies heavily on primary care provider recognition of clinical risk factors. Primary care providers may lack awareness of the ubiquity of osteoporosis and often prioritize acute over preventive health care due to competing demands. However, given the strong predictive value of prior fracture on future fracture, it is critical that we develop approaches to primary prevention. Risk identification tools that incorporate knowledge of diseases, medications, and other clinical risk factors for osteoporosis are needed to effectively provide primary fracture prevention in a systematic fashion.^(^ ^3^, ^4^ ^)^ The Rural Bone Health Team (BHT) used a telehealth clinic model to deliver primary prevention to rural veterans receiving care from the Veterans Health Administration (VHA) in the Mountain West region of the United States.^(^ ^7^ ^)^

The BHT model of care is described in detail elsewhere,^(^ ^7^ ^)^ but in brief, BHT is a primary prevention model that reviews electronic health record data to identify veterans receiving VHA primary care who have not been evaluated or treated for osteoporosis. BHT directly communicates with at‐risk veterans to arrange for DXA, reviews DXA results and conducts clinical assessments, and provides bone health care directly to veterans by telephone, with notes to veterans' primary care providers in the electronic health record. BHT is directed by a rheumatologist (KLM) with osteoporosis expertise and supported by program support assistants, clinical nurses operating under evidence‐based protocols, and advanced practice providers (i.e., a physician assistant and a clinical pharmacist). Veterans with osteoporosis risk are identified using a clinical dashboard populated with data from the Veterans Health Administration (VHA) Corporate Data Warehouse (CDW) data on age (e.g., for women ≥ 65; men ≥ 80), evidence of exposure to chronic glucocorticoid therapy (i.e., prednisone), androgen deprivation therapy (i.e., leuprolide), or aromatase inhibitors (i.e., anastrozole), and an Osteoporosis Self‐Assessment Tool (OST)^(^ ^8^, ^9^, ^10^, ^11^ ^)^ score of ≤1. Veterans with evidence of these risks were invited to participate in BHT regardless of whether they had evidence of recent osteoporosis management (i.e., DXA or bisphosphonate). Although veterans engaged in osteoporosis care among this cohort were a minority, the clinical BHT wanted to provide equitable access to the same high‐quality, virtual care for all rural veterans and their primary care teams.

Our prior work demonstrated implementation feasibility from the perspective of VHA primary care providers (PCPs) and patients, with a quarter of identified rural veterans accepting DXA screening and 91% of those eligible choosing to initiate fracture risk–reducing therapies.^(^ ^12^ ^)^ Qualitative interviews with veterans invited to enroll in BHT revealed the role of competing comorbidities and beliefs about perceived osteoporosis importance and susceptibility on patient decision making around DXA.^(^ ^13^ ^)^ Patient medication decision making was found to stem from their desire to preserve of quality of life and maintain function and from concerns about side effects in the context of aging, physical limitations, or polypharmacy.^(^ ^13^ ^)^ Interviews with VHA PCPs indicated that PCPs believed that the BHT provided a higher quality of osteoporosis care than could be provided in primary care setting without increasing workload to primary care teams. However, PCPs expressed a desire for more feedback from the BHT on PCPs' osteoporosis care, education on osteoporosis management, and increased visibility of the BHT service.^(^ ^14^ ^)^ Although prior research demonstrated that the BHT was feasible, clinic enrollment was low (22%). The low enrollment presented an opportunity to improve clinic reach to serve patients who declined BHT care. The objective of this study was to identify social determinants of health and clinical factors associated with enrollment in the BHT program and with osteoporosis medication initiation and to inform the understanding of implementation barriers to primary prevention programs.

## Materials and Methods

### Study design and data sources

This study received Institutional Review Board (IRB) approval from the University of Iowa (IRB #201807728). The study was conducted using data from the CDW, which is a repository of administrative, demographic, and clinical data. Data from the following CDW domains were used: outpatient, inpatient, patient, and enrollment. Other data sources included: decision support system (DSS) pharmacy, planning systems support group (PSSG) geocoded, and vital status. The CDW inpatient and outpatient tables included all patients admitted to VHA healthcare facilities with data on primary diagnosis using the International Classification of Diseases, 10th revision diagnosis codes (ICD‐10) and procedures performed during a visit using Current Procedural Terminology (CPT) codes. Medications filled at VHA pharmacies were obtained from the VHA National Data Extract Pharmacy data set. The vital status file included date of birth, date of death, and veteran eligibility. Marital status and means tests were obtained from the SPatient file, and race was determined using the patient race file. BHT note templates with embedded structured data labels in the electronic health record (EHR) supported a customized information management system that enhanced point‐of‐care decision making and allowed for tracking of patient care delivery information such as risk factors, diagnoses, and treatments. Data known as “health factors” were derived from those templates. Patient data from each of the aforementioned sources were associated with unique patient identifiers that allowed all data sets to be merged.

### Study population

**Fig. 1:**
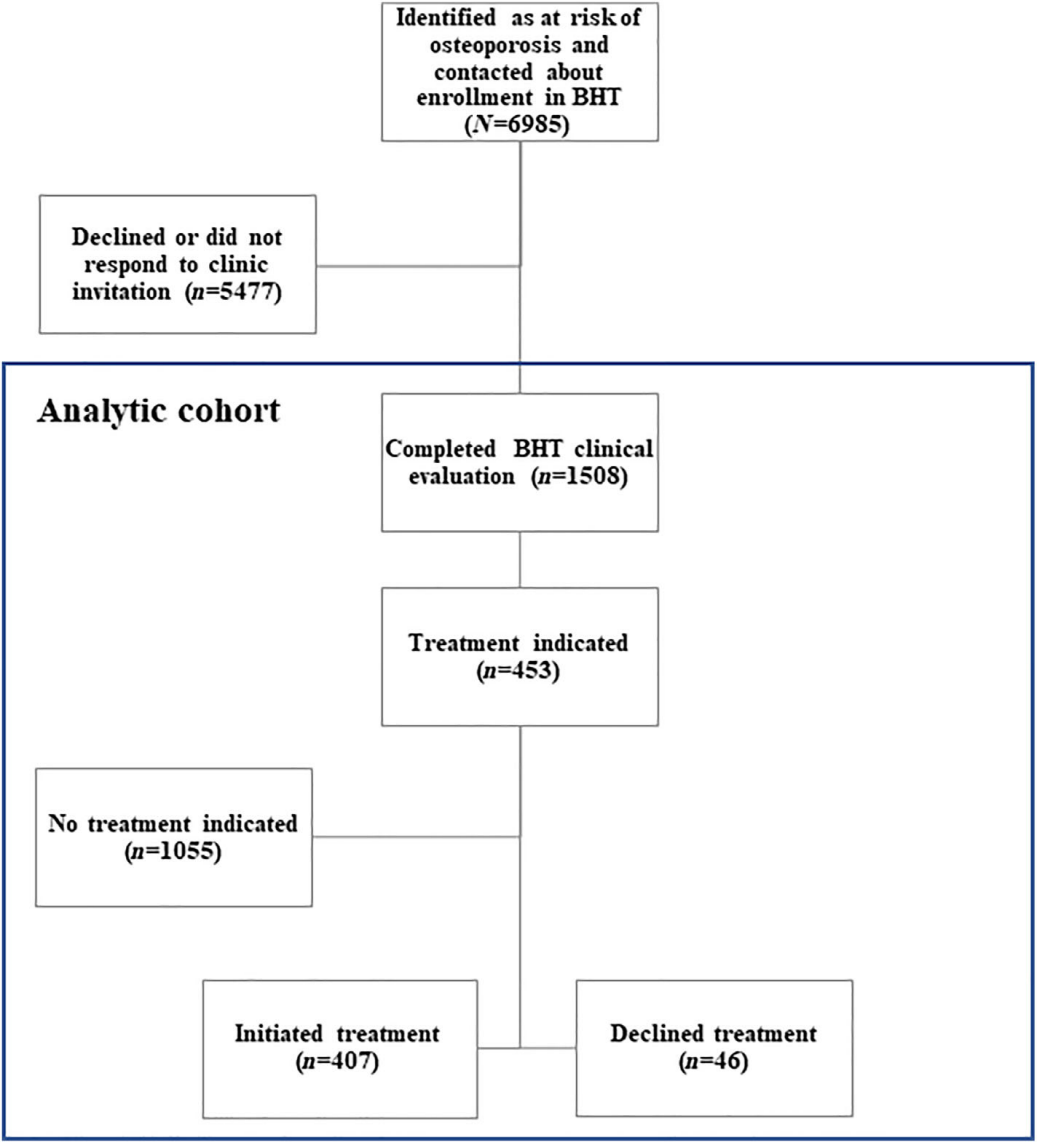
Overview of clinical and analytic cohort.

We used national VHA data for fiscal years 2014 to 2018 to identify veterans aged 50 to 99 who were invited to enroll in the BHT program. The cohort consisted of 6,985 veterans, 1,508 patients who enrolled and received DXA, and 5,477 veterans who declined care or never responded to the clinic's invitation. There were 453 veterans for whom medication was indicated and 407 who initiated treatment. See Figure 1 for an overview of cohort development.

### Outcomes and study variables

Our primary objective was to identify factors associated with BHT enrollment; a secondary objective was to identify factors associated with medication initiation. We did not use a priori hypothesis testing in this observational study but utilized existing clinical data to evaluate the acceptability of the BHT model. Variable selection was informed by clinical expertise and review of the literature on social determinants of preventive health care (e.g., gender, race, distance from healthcare facility, cost, age) and clinical factors that might influence perceived risk and importance (e.g., comorbidity or disease severity). Independent variables included gender, race, age, marital status, socioeconomic status as defined by the VHA means test, distance from a VHA DXA facility, number of primary care encounters within the past year, and common clinical factors associated with fracture in veterans (i.e., prior fracture, androgen deprivation therapy (ADT), corticosteroid medication, rheumatoid arthritis (RA), Parkinson's disease, and a weighted comorbidity index).^(^ ^15^ ^)^ Age was categorized as 50–64, 65–79, 80–94, and 95–99. A dichotomous variable was constructed for distance between residence and nearest DXA with separate groups used to represent veterans who traveled 0–40 miles versus >40 miles. Given the small number of veterans reporting race other than white in the cohort, race was categorized as white, other, and unknown. Disease severity was measured using the van Walraven comorbidity index, a weighted score of 30 comorbidities.^(^ ^15^ ^)^ For the medication analysis, we also evaluated several additional factors collected as part of the DXA process of care, including parental history of hip fracture, prior history of smoking, current smoking, weight bearing exercise, fall risk, and osteopenia or osteoporosis diagnosis.

### Statistical analysis

Data were first examined for outliers and completeness. Descriptive statistics, including percentages, means, medians, and interquartile ranges (as appropriate), were calculated for gender, race, age, marital status, means test, distance to nearest DXA, primary care encounter within the past year, prior fracture, androgen deprivation therapy (ADT), corticosteroid medication, RA, Parkinson's disease, van Walraven comorbidity index, parental history of hip fracture, prior history of smoking, current smoking, weight bearing exercise, fall risk, osteopenia, and osteoporosis.

To determine the predictors of enrollment in the BHT program, bivariate regression models were constructed using generalized estimating equations with a logit link function. This method accounted for clustering of patients within hospitals. All variables with *p* < 0.25 in bivariate regression models were included in fully saturated model. Backward regression was then utilized and all variables significant at *p* < 0.05 were retained in a final reduced model.

Since the number of veterans for whom osteoporosis medication treatment was indicated was small and individual cell counts were sparse, crosstabs were generated and the Fisher's exact test or the chi‐square test was used to determine group differences between those who chose to initiate medication and those who declined. All statistical analyses were performed on VINCI using SAS Enterprise Guide version 8.2 (Cary, NC).

## Results

For the 27‐month study period, 6,985 rural veterans were identified as having osteoporosis risk and were contacted by mail. See Table 1 for cohort characteristics. Of those contacted, 22% enrolled in the BHT clinic. Enrollees were predominantly male (94%), white (93%), married (68.5%), and copayment exempt (53%), with a mean age of 76. Only 2.9% of enrollees had undergone prior DXA testing, 1.1% had received prior osteoporosis medication, 1.8% had an osteoporosis diagnosis, 1.7% used androgen deprivation therapy, and 0.13% had a history of any fracture, whereas 31.6% had previously used corticosteroids. In our regression model, factors associated with a lower likelihood of enrolling in BHT included age greater than 80, living more than 40 miles from VHA services, single or widowed marital status, out‐of‐pocket copayment requirement, preexisting fragility fracture, and diagnosis of RA (Table 2). Of the 1,508 veterans who enrolled in the BHT program, 30% (453) had treatment indications. Most veterans with treatment indication had osteopenia with high fracture risk, were male and white, and had an average age of 77 (Table 3). Few of these veterans had previously received DXA (5.3%) or osteoporosis medication (2.2%) and over one‐third had a history of prior corticosteroid use (Table 3). Analysis of differences between those rural veterans who initiated and those who declined medication to prevent fragility fracture indicated that veterans who started medication also were more likely to have had a history of two or more falls in the prior year and a parental history of fracture, to have been current smokers, and to have reported that they had engaged in weight‐bearing exercise (Table 3).

**Table 1:** Sociodemographic and Clinical Characteristics of Eligible Veterans by Decision to Enroll

	Enrolled in BHT (*N* = 1508) Percent (*N*)	Declined care or never responded (*N* = 5477) Percent (*N*)
Sociodemographic characteristics		
Age		
50—64	12.33 (186)	8.86 (485)
65—79	57.29 (864)	49.04 (2,686)
80—99	30.37 (458)	42.10 (2,306)
Distance between veteran residence and nearest VHA hospital		
0–40 miles	12.40 (187)	6.41 (351)
> 40 miles	87.60 (1,321)	93.59 (5,126)
Sex (male)	94.16 (1,420)	93.79 (5,137)
Race		
White	94.96 (1,432)	92.06 (5,042)
Indigenous, American Indian, Alaska Native	0.66 (10)	0.86 (47)
African American	0.53 (8)	0.31 (17)
Native Hawaiian	0.27 (4)	0.47 (26)
Asian	0.27 (4)	0.24 (13)
Unknown	3.32 (50)	6.06 (332)
Marital status		
Married	68.50 (1,033)	62.50 (3,423)
Single	23.94 (361)	26.40 (1,446)
Widowed	7.29 (110)	10.88 (596)
Unknown	0.27 (4)	0.22 (12)
VHA means category		
Copayment exempt	53.45 (806)	54.28 (2,973)
Copayment required	26.99 (407)	30.42 (1,666)
Unknown	19.56 (295)	15.30 (838)
Preexisting clinical		
DXA	2.92 (44)	2.92 (160)
Osteoporosis medication	1.19 (18)	2.76 (151)
Any fracture (i.e., hip, pelvis, rib, vertebral, wrist)	0.13 (2)	0.57 (31)
Androgen deprivation therapy	1.66 (25)	2.41 (132)
Corticosteroid medication use	31.63 (477)	30.64 (1,678)
Back pain	15.90 (240)	13.24 (725)
Rheumatoid arthritis	0.66 (10)	0.93 (51)
Osteoporosis	1.79 (27)	2.23 (122)
Parkinson's disease	1.19 (18)	1.15 (63)
Comorbidity index (mean, SD)	2.44 (4.97)	2.83 (5.53)
Number of primary care encounters during previous year	30.04 (453)	31.09 (1,703)

**Table 2:** Factors Associated with BHT Enrollment, Multivariate Regression Model

	Fully saturated model OR (95% CI)	*p* value	Reduced model OR (95% CI)	*p* value
Age (ref: 48—64)				
65—79	0.87 (0.72–1.05)	0.152	0.89 (0.73–1.10)	0.28
80—99	0.54 (0.42–0.71)	<.0001	0.57 (0.45–0.72)	<0.0001
Distance between veteran's residence and nearest VHA hospital				
	0.54 (0.43–0.67)	<.0001	0.54 (0.43–0.68)	<0.0001
Gender (ref: male)	0.79 (0.57–1.10)	0.166		
Race (ref: white)				
Other	0.80 (0.51–1.25)	0.318	0.79 (0.51–1.24)	0.31
Unknown	0.57 (0.37–0.86)	0.008	0.57 (0.38–0.84)	0.005
Marital status (ref: married)				
Single	0.73 (0.60–0.90)	0.003	0.72 (0.57–0.91)	0.005
Widowed	0.70 (0.58–0.85)	0.0002	0.69 (0.56–0.84)	0.0003
Unknown	1.11 (0.39–3.13)	0.841	1.11 (0.38–3.27)	0.848
VHA means category (ref: copayment required)				
Copayment exempt	1.13 (1.03–1.25)	0.013	1.13 (1.02–1.25)	0.015
Unknown	1.39 (1.20–1.62)	<.0001	1.33 (1.07–1.66)	0.011
Clinical factors				
Any fracture (i.e., hip, pelvis, rib, vertebral or wrist)	0.25 (0.12–0.50)	<.0001	0.24 (0.10–0.56)	0.0009
Androgen deprivation therapy	0.63 (0.40–0.99)	0.047		
Corticosteroid medication use	1.02 (0.87–1.20)	0.787		
Rheumatoid arthritis	0.65 (0.49–0.87)	0.004	0.68 (0.51–0.90)	0.007
Parkinson's disease	1.12 (0.67–1.87)	0.673		
Comorbidity index	0.99 (0.98–1.00)	0.187		

**Table 3:** Factors Associated with Medication Initiation Among BHT Patients

	Medication indicated (*N* = 453)			
	Initiate (*N* = 407) % (*n*)		Decline (*N* = 46) % (*n*)	
Sociodemographic characteristics				
Age				
50—64	5.65	(23)	4.35	(2)
65—79	48.65	(198)	60.87	(28)
80—99	45.70	(186)	34.78	(16)
Distance between veteran's residence and nearest VHA DXA clinic				
0–40 miles	7.86	(32)	8.70	(4)
> 40 miles	92.14	(375)	91.30	(42)
Male	96.56	(393)	93.48	(43)
Race				
White	95.82	(390)	95.65	(44)
Other	0.98	(4)	0	(0)
Unknown	3.19	(13)	4.35	(2)
Marital status				
Married	66.09	(269)	73.91	(34)
Single	24.57	(100)	17.39	(8)
Widowed	8.60	(35)	8.70	(4)
Unknown	0.74	(3)	0	(0)
VHA means category				
Copayment exempt	56.02	(228)	52.17	(24)
Copayment required	27.27	(111)	28.26	(13)
Unknown	16.71	(68)	19.57	(9)
Clinical factors prior to BHT enrollment				
Prior DXA	4.91	(20)	8.70	(4)
Prior osteoporosis medication	1.72	(7)	6.52	(3)
Prior androgen deprivation therapy	1.23	(5)	0	(0)
Prior corticosteroid medication use	32.19	(131)	34.78	(16)
Parkinson's disease	1.97	(8)	0	(0)
Rheumatoid arthritis	1.47	(6)	0	(0)
Comorbidity index (mean, SD)	3.06	(5.37)	3.28	(5.39)
Clinical factors identified through BHT screening				
Parental history of hip fracture	97.30*	(396)	89.13	(41)
Prior history of smoking	39.80	(158)	36.59	(15)
Current smoking	17.13^*^	(68)	2.44	(1)
Weight bearing exercise	94.35^*^	(384)	78.26	(36)
History of two or more falls in prior year	18.67^*^	(76)	2.17	(1)
BHT diagnoses for treatment indication				
Osteopenia with low fracture risk	0.49	(2)	2.17	(1)
Osteopenia with high fracture risk	57.49	(234)	45.65	(21)
Osteoporosis by DXA	36.12	(147)	46.65	(21)
Osteoporosis by clinical fracture	9.83	(40)	6.52	(3)

*Fisher's exact test or the Chi‐square test was used to determine group differences between those who chose to initiate medication and those who declined. The asterisk denotes statistical significance at *p* < 0.05.

## Discussion

The BHT model is a feasible approach to primary prevention in an integrated healthcare system, interpreting DXA for a significant number of previously unscreened rural veterans with osteoporosis risk. However, prior to broader implementation, assessment of factors associated with patient refusal of DXA was warranted. In this study we demonstrated that reducing reliance on primary care providers to initiate DXA orders is important but insufficient to address the epidemic of underscreening. Social determinants of health and health behaviors likely play a complex, interdependent role in patient decision making to accept DXA referral.

The cost of osteoporosis care in the VHA is generally low for veterans who have a copayment. At the time of this study, DXA copayment was US$25 and monthly oral alendronate was US$7. However, even at this low cost, copayment persists as a limitation to DXA completion for some veterans. Clinic design integrated veterans' eligibility for DXA from non‐VHA providers through the Mission Act,^(^ ^14^ ^)^ which allows veterans to obtain DXA at the nearest location in their community. Yet for this population of rural veterans, the closest DXA may be more than 60 miles away—regardless of whether the DXA center is in the VHA or private sector— making travel during the winter months challenging or distances too burdensome. It is feasible that as new risk identification tools are validated for various populations, older rural veterans at high risk for fragility fractures could benefit from fracture risk–reducing therapies without first undergoing DXA. Indeed, some countries recommend this approach to fracture prevention for those with high risk of fracture in whom a DXA would not significantly change their risk category.^(^ ^15^, ^16^, ^17^ ^)^ However, even if this approach were used at a population level, most would fall into a moderate risk category where DXA would be recommended to determine treatment eligibility by incorporating bone mineral density into the fracture risk prediction.

Beyond cost and transportation challenges, in this sample, married veterans and those aged 65–79 were more likely to enroll in the BHT program than those who were unmarried or older (80–99). These findings are consistent with other demographic research that suggests married persons may have better bone health, general health, and mortality outcomes, particularly men.^(^ ^18^, ^19^, ^20^, ^21^ ^)^ Though older adults are at higher risk of osteoporosis and fracture, they are also more likely to accumulate psychological and social deficits of frailty, beyond medical comorbidities, that are barriers to engagement in their health care.^(^ ^22^ ^)^ Our qualitative interviews with rural veterans who enrolled or declined enrollment in the BHT program suggested that competing comorbidities combined with varying perceptions about osteoporosis susceptibility and importance contributed to decisions to engage in screening.^(^ ^13^ ^)^ In the current study, veterans with a preexisting fragility fracture and RA were also less likely to enroll and receive screening, despite their higher risk for future fractures. It is possible that these veterans had previously declined medications or represent a frailer population with greater barriers to engagement that are not measured in our model. DXA screening for osteoporosis in men and veterans is known to be low,^(^ ^23^ ^)^ even in those with strong risk factors such as a prior fragility fracture^(^ ^24^, ^25^ ^)^; however, a limitation of our study is that we cannot ascertain medication refusal in the administrative data. Interestingly, studies have indicated that most patients who have sustained a fragility fracture in adulthood remain in the precontemplative stage of change with regard to osteoporosis management^(^ ^26^, ^27^ ^)^ and may not link their fracture to an osteoporosis diagnosis or need for treatment.^(^ ^28^ ^)^ Regarding the association of RA with DXA refusal, we hypothesize that these patients may prefer to pursue or discuss DXA screening with their usual rheumatologist; however, this is unknown.

Initiation of osteoporosis treatment among BHT patients was quite high (91%). This finding stands in contrast to rates of treatment uptake of 3%–9% observed in other primary prevention interventions that use mail invitations for DXA.^(^ ^29^, ^30^, ^31^ ^)^ However, these interventions relied on the PCP or their patients to initiate screening and on the PCP to initiate treatment, rather than a dedicated specialty care team. Evidence suggests that having a DXA is a predictor of treatment adherence,^(^ ^32^, ^33^ ^)^ and so it is possible that those patients who agree to DXA screening are already more likely to agree to osteoporosis treatment. Of note, a study of factors associated with readiness to initiate osteoporosis treatment in women at high risk of fracture found that being aware of the diagnosis was significantly associated with a contemplative state of readiness.^(^ ^26^ ^)^ It is conceivable that undergoing a DXA test increases the likelihood of a diagnosis being made and discussed, which may increase the likelihood of starting treatment. Similarly, in our related qualitative study of patient rationale for DXA refusal, some evidence suggests that patients who strongly oppose initiating medication will also refuse DXA.^(^ ^34^ ^)^

In our analysis of factors associated with treatment initiation, patients with a history of falls in the last year, parental history of fracture, report of current smoking, and report of participation in weight‐bearing exercise were more likely to initiate treatment. Although earlier studies in women showed an increase in osteoporosis treatment uptake or adherence among those with higher fall risk,^(^ ^35^ ^)^ qualitative examinations of patient experience and perception suggest that patients often do not connect their falls with fracture risk. Similarly, parental history of fracture in women has been found to be associated with improved osteoporosis treatment uptake and adherence,^(^ ^36^, ^37^ ^)^ but this factor is notoriously difficult to capture due to inconsistent documentation in the electronic health record, and data examining this factor in men or veterans are scarce. Surprisingly, current smokers were more likely to initiate osteoporosis treatment. This finding contradicts adherence studies in osteoporosis and other health conditions that indicate smoking as a risk factor for low treatment uptake or nonadherence.^(^ ^32^, ^33^, ^38^, ^39^, ^40^ ^)^ However, these veterans may represent a population more likely to adhere owing to their completion of DXA and may see osteoporosis medication as an opportunity to improve bone health and counteract the effect of smoking. Alternatively, since smoking is more prevalent among veterans, it may not be as strong a proxy for health behavior as it is in the general population. The association of exercise with treatment initiation is consistent with studies on osteoporosis^(^ ^41^ ^)^ and other conditions,^(^ ^42^, ^43^ ^)^ demonstrating that participation in exercise is associated with treatment adherence.

Our study has several limitations worth noting. We report on a regional clinic in the Mountain West serving rural veterans, and barriers to care for urban veterans may differ. Veterans for whom we could not ascertain race were less likely to enroll in the BHT program, and the meaning of this finding remains uncertain. Similarly, we may be underpowered to detect racial differences in BHT enrollment, owing to the small number of racial minority veterans residing in the enrollment area and the proportion of veterans for whom we were unable to ascertain race.^(^ ^44^ ^)^ As an integrated single‐payer system, VHA reduces many barriers to health equity, yet known racial disparities exist in access to care and clinical outcomes. Most germane to this cohort, veterans who identify as Indigenous or American Indian are more likely than other racial minority populations to have missing race or incomplete data in VHA records. The number of veterans refusing fracture‐reducing therapy was small, which could reduce our power to detect factors associated with nonadherence. Our results are limited to data available through care provided at the VHA, and although BHT program enrollment was limited to veterans who are known users of VHA primary care, some veterans invited to enroll in BHT may rely on private‐sector healthcare providers for their specialty care.

One of the major strengths of this study is that our BHT cohort represents both a unique and understudied population. Additionally, this study represents one of very few primary prevention or population health models for bone health in the literature, despite evidence that risk identification strategies may be a more cost‐effective approach to bone health in men.^(^ ^23^, ^45^, ^46^, ^47^, ^48^, ^49^ ^)^ The notable proportion of men enrolled in this primary prevention program provides a special opportunity for understanding implementation barriers in a population that is widely understood to have low perceived severity and susceptibility to osteoporosis. We found that an impressively high proportion of veterans eligible for fracture risk–reducing therapies chose to initiate therapy, and efforts to identify and alleviate barriers to screening may represent an effective pathway to improving bone health in this population. Future efforts should include examining the impact of this population approach to bone health on fracture outcomes and medication adherence. However, despite the demonstration of feasibility, our study was potentially underpowered to identify the contribution of race, gender, and ethnicity to program acceptability. Future studies in a more diverse population should examine clinical and social factor impact access to bone health care and perceptions of risk, as well as how healthcare systems can incorporate clinical and social predictors of bone health engagement to improve bone health care across a variety of clinical settings.

## Author Contributions

**Karla L. Miller:** Conceptualization; data curation; investigation; writing – original draft; writing – review and editing. **Kimberly McCoy:** Formal analysis; methodology; writing – original draft; writing – review and editing. **Chris Richards:** Data curation; software; writing – original draft. **Aaron Seaman:** Conceptualization; writing – original draft. **Samantha L. Solimeo:** Conceptualization; investigation; methodology; writing – original draft, writing – review & editing.

## Disclosures

All authors are employees of the VHA. K.L. Miller has no potential conflicts of interest to disclose. K.D. McCoy has no potential conflicts of interest to disclose. C.C. Richards has no potential conflicts of interest to disclose. A.T. Seaman has no potential conflicts of interest to disclose. S.L. Solimeo has no potential conflicts of interest to disclose.

## Funding

This work was supported by grants from the Department of Veterans Affairs Office of Rural Health Veterans Rural Health Resource Center‐Iowa City (03468 to S.L. Solimeo). S.L. Solimeo received support from the VHA Health Services Research & Development (HSR&D) Comprehensive Access & Delivery Research and Evaluation Center (CADRE), Iowa City VHA Health Care System, Iowa City, IA (Award CIN 13–412). S.L. Solimeo received support from VHA HSR&D (Award CDA 13–272). The VHA had no role in the analysis or interpretation of data or the decision to report these data in a peer‐reviewed journal. The views expressed in this article are those of the authors and do not necessarily reflect the position or policy of the VHA or the United States government.

### Peer Review

The peer review history for this article is available at https://publons.com/publon/10.1002/jbm4.10682.

## Data Availability

The data that support the finding of this study are not openly available due to human subject regulations associated with our VHA data.
